# Nondestructive Characterization by Advanced Synchrotron Light Techniques: Spectromicroscopy and Coherent Radiology

**DOI:** 10.3390/s8128378

**Published:** 2008-12-16

**Authors:** Giorgio Margaritondo, Yeukuang Hwu, Jung Ho Je

**Affiliations:** 1 Ecole Polytechnique Fédérale de Lausanne (EPFL), CH-1015 Lausanne, Switzerland; 2 Institute of Physics, Academia Sinica, Nankang, Taipei, Taiwan; E-Mail: phhwu@sinica.edu.tw; 3 Institute X-ray Imaging Center, Department of Materials Science and Engineering, Pohang University of Science and Technology, Pohang, 790-784, South Korea; E-Mail: jhje@postech.kr

**Keywords:** synchrotron, photoemission, radiology

## Abstract

The advanced characteristics of synchrotron light has led in recent years to the development of a series of new experimental techniques to investigate chemical and physical properties on a microscopic scale. Although originally developed for materials science and biomedical research, such techniques find increasing applications in other domains – and could be quite useful for the study and conservation of cultural heritage. Specifically, they can nondestructively provide detailed chemical composition information that can be useful for the identification of specimens, for the discovery of historical links based on the sources of chemical raw materials and on chemical processes, for the analysis of damage, their causes and remedies and for many other issues. Likewise, morphological and structural information on a microscopic scale is useful for the identification, study and preservation of many different cultural and historical specimens. We concentrate here on two classes of techniques: in the first case, photoemission spectromicroscopy. This is the result of the advanced evolution of photoemission techniques like ESCA (Electron Microscopy for Chemical Analysis). By combining high lateral resolution to spectroscopy, photoemission spectromicroscopy can deliver fine chemical information on a microscopic scale in a nondestructive fashion. The second class of techniques exploits the high lateral coherence of modern synchrotron sources, a byproduct of the quest for high brightness or brilliance. We will see that such techniques now push radiology into the submicron scale and the submillisecond time domain. Furthermore, they can be implemented in a tomographic mode, increasing the information and becoming potentially quite useful for the analysis of cultural heritage specimens.

## 1. Introduction

The defense and preservation of cultural heritage can greatly benefit from the development of new techniques for analysis – in particular, non-destructive methods. In recent years, some of the most promising [1-6] have been based on the unique characteristics of the x-ray and ultraviolet radiation emitted by synchrotron sources of the “storage ring” class [7, 8].

In most cases, the use of a synchrotron source makes it possible to combine the advanced performance of standard spectroscopic and imaging methods with a high level of spatial and/or time resolution [9-11]. The developments in this domain are so fast that many potential users remain unaware of them – and thus do not profit from their unique performances to solve problems that are otherwise difficult or impossible to tackle.

This issue is particularly relevant for colleagues involved in the study and preservation of cultural heritage. In many cases, nondestructive analysis of the chemical, morphological and structural properties on a microscopic scale can assist the identification, analysis and conservation of cultural and historical specimens. For example, examination of the pigments in antique pottery or paintings can certify the authenticity of specimens by linking them to specific chemical mines and/or to chemical processes developed in different historical periods. Detailed nondestructive microscopic analysis of the chemical properties and morphology can identify early symptoms of deterioration, study their causes and suggest possible remedies.

**Figure 1.**
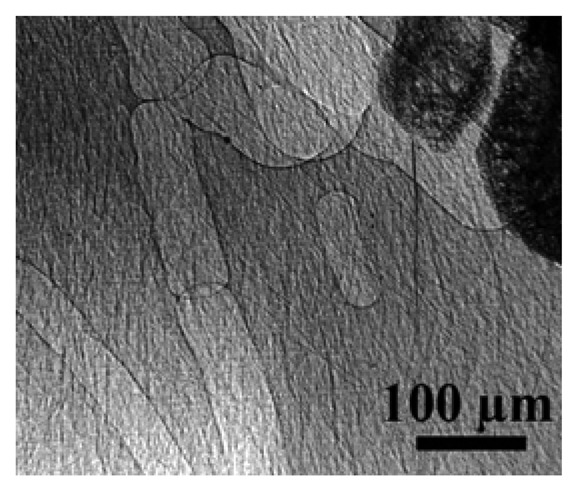
An example of the advanced capabilities of microradiology with spatially coherent synchrotron x-rays: the area near the ankle of a mouse, taken without any contrast agent [12] and revealing very small details. The image recording time was 30 milliseconds.

Such examples and many others suggest that the solution of many problems concerning cultural heritage could greatly profit from a broader dissemination of the advances of synchrotron techniques -- that were particularly spectacular in recent years. Just to provide a first example, Figure 1 shows [12] what is possible today with the synchrotron technique known as coherent-x-ray microradiology. The comparison with standard radiology is striking: unprecedented resolution is achieved in revealing small structural details. As discussed in detail later, such a technique is only possible with a synchrotron source and specifically requires the characteristic known as “spatial coherence” or “lateral coherence” [8, 10, 11].

The objective of the present short review is to present two non-destructive synchrotron analysis techniques in a form accessible to non-specialists, but sufficient to appreciate the potential applications and to conceive the outline of experiments. The first technique is photoemission spectromicroscopy, the combination of photoemission spectroscopy and high lateral resolution [8, 9]. The second is microradiology with coherent x-rays, whose power is emphasized by Figure 1. We will not present an exhaustive review of experiments with these techniques. The selected examples of applications are meant to illustrate what can be done with these techniques and hopefully inspire new applications in the art and cultural domains.

## 2. Synchrotron Light, Brightness and Coherence

The essential ingredient for both of the techniques discussed here is synchrotron light, with its high brightness and lateral coherence [7, 8]. We thus start our discussion with a brief introduction to the properties of synchrotron light and their link to Einstein's relativity theory.

The term “synchrotron light” refers to the electromagnetic radiation – x-rays, ultraviolet, visible and infrared – emitted by high-energy electrons circulating in a particle accelerator. Specifically, the accelerators used in modern facilities [7] are “storage rings” in which the electron, after being injected and reaching their final energy, circulate under ultrahigh vacuum for hours or days emitting synchrotron light – that is collected by several beamlines and used for different experiments in parallel.

**Figure 2.**
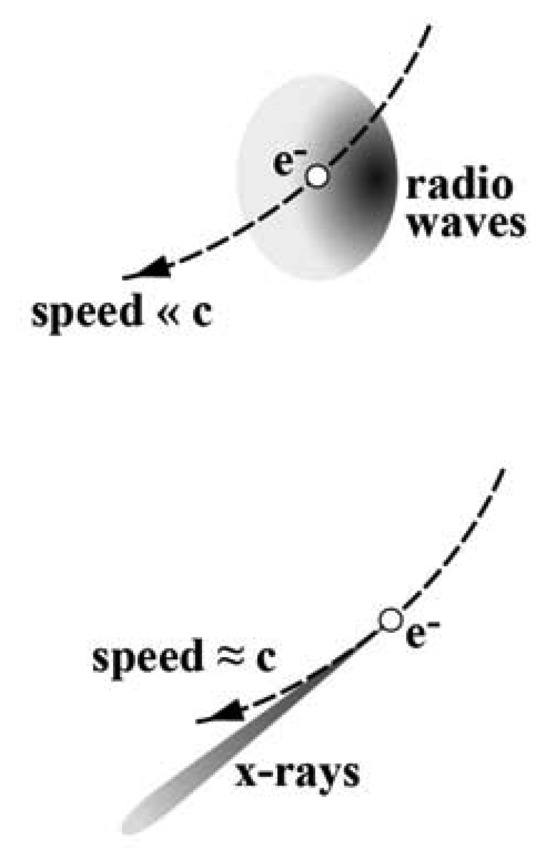
Synchrotron light emission in the nonrelativistic (top) and relativistic cases. When the electron speed in the storage ring approaches the speed of light, the emission is strongly peaked in the forward direction and centered in the x-rays region rather than consisting of radio waves.

In a storage ring, bending magnets keep the electrons in a fixed trajectory by centripetally accelerating them. The electrons are charged particles that, when accelerated, emit electromagnetic waves. For electrons with nonrelativistic energy, the emission would consists of radio waves and occur over a wide angular range. On the contrary, as the electron energy reaches relativistic levels, the emission becomes peaked in the forward direction of the motion and the electron behaves as a “torchlight” – see Figure 2 [7, 8]. Furthermore, the emission is no longer confined to radio waves but spread over a broad frequency band centered in the x-ray domain.

**Figure 3.**
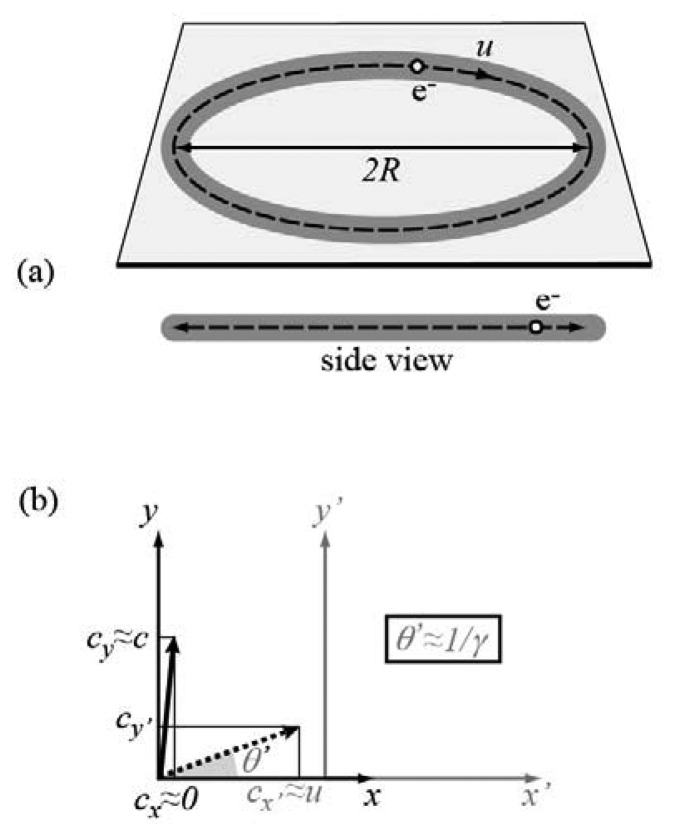
Schematic explanation of the “torchlight” emission of synchrotron light. Top: an electron circulating in a storage ring, when seen from the side, looks like a charge oscillating in a linear antenna. For nonrelativistic electrons, the emission occurs over a broad angular range and is centered in the x-ray domain. Bottom: the Lorentz velocity transformation squeezes the broad angular emission range in the electron frame to a very narrow cone in the laboratory frame.

All of the above facts can be easily understood, but a full theoretical treatment would require some complex mathematics. We will adopt instead a simplified discussion. Consider first (Figure 3a) the case of nonrelativistic electrons and imagine, for simplicity, an electron of speed *u* ≪ *c* moving along a circular path of radius *R*. Observed from the side and from its plane, the circular trajectory looks like a line and the circulating electron like an oscillating charge along a linear antenna. This charge emits a broad angular radiation pattern with characteristic frequency *u*/(2π*R*). For *R* in the range of meters (and *u* ≪ *c*), this frequency is in the radio wave range.

Let us now consider the relativistic case. Figure 3b explains the “torchlight effect”. The emission in the electron reference frame (*x*, *y*) occurs again over a broad angular range. However, it becomes forward-peaked after Lorentz transformation to the laboratory frame (*x'*, *y'*). Take in fact a photon emitted in the electron frame in a direction (angle *θ*) almost perpendicular to the electron motion (there is no emission in the perpendicular direction). The velocity components of the photon in the electron frame are *c_x_≈* 0, *c_y_≈ c* (giving of course to a speed *c*). In the laboratory frame, the Lorentz velocity transformation gives *c_x'_* ≈ *u.* Since the speed must remain equal to *c*, the laboratory-frame emission angle *θ'* equals cos^-1^(*c*/*c_x'_*) ≈ cos^-1^(*c*/*u)*. If *u* → *c* and therefore *θ'* is small, then cos(*θ'^2^*) ≈ (1 - *θ*'*^2^*/2) ≈ *c*/*u* and *θ' ≈* [2(1*-u*/*c)*]*^1/2^=* [2(1*-u^2^*/*c^2^*)/(1+*u*/*c*)]*^1/2^≈* 1/*γ*. Thus, the synchrotron light emission occurs over an angular range of the order of 1/*γ.* If the electron energy is of the order of gigaelectronvolts (GeV), typical of a storage ring, then 1/*γ <* 0.5 milliradian.

**Figure 4.**
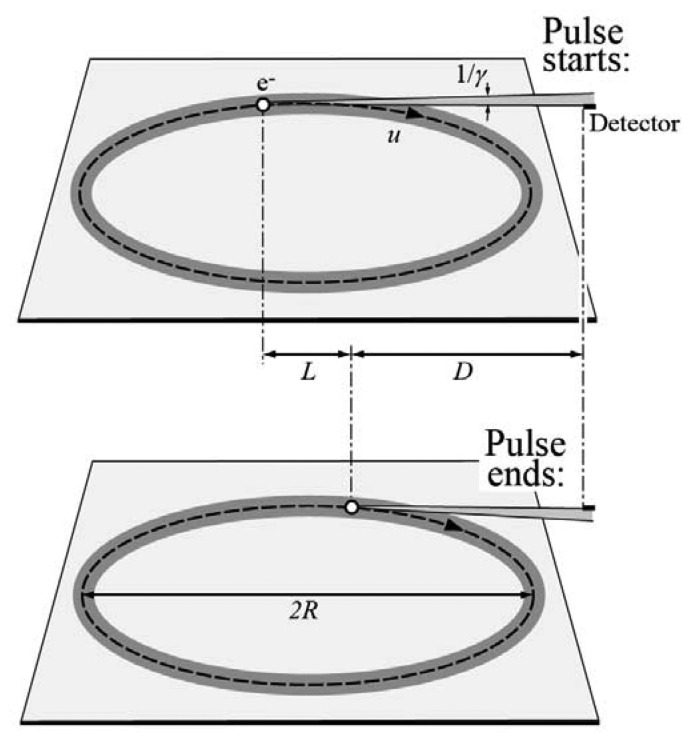
Schematic illustration of how the “torchlight” effect and relativity explain the fact that synchrotron light is spectrally centered in the x-ray domain.

Relativity and the “torchlight effect” also explain [7, 8] the spectral emission changes from radio waves to a broad band extending to the x-rays. Consider (Figure 4) an electron circulating around the ring with its “torchlight” emission and a small-area detector at a distance *D*. This electron illuminates the detector only once for every turn, during a very short time Δ*t*.

The top part of Figure 4 illustrates the position of the electron when it emits the photons corresponding to the beginning of Δ*t* and the bottom part the position for its end. The two positions are separated by an arc of trajectory corresponding to an angle ≈1/*γ*, whose length is *L* ≈ *R*(1/*γ*). The beginning of Δ*t* occurs *≈*(*D* + *L*)/*c* seconds after the emission of the corresponding photons and ends ≈*L*/*u̲* + *D*/*c* seconds later. Therefore, Δ*t* ≈ (*R*/*γ*) (1/*u* − 1/ *c)*. For *u* → *c*, we have (1/*u* − 1/*c*) = (1/*u*) (1 − *u^2^*/*c^2^*)/(1 + *u*/*c*) ≈ 1/(2*cγ*^2^), and Δ*t* ≈ *R*/(2*cγ*^3^).

The Fourier frequency spectrum corresponding to this series of short pulses is a broad band whose bandwidth is determined by 1/Δ*t* ≈ 2*cγ*^3^/*R*. The corresponding photon energy bandwidth, of the order of 2*hcγ*^3^/*R*, extends to the x-rays. For example, 1 GeV electrons with *R* = 1 m would correspond to 2*hcγ*^3^/*R* ≈ 20 keV.

Synchrotron light is very intense. This is due in part to the emission mechanism, but also to the fact that the emitting electrons are not part of solids or liquids so that there is no risk of damage of the medium when the intensity is too high.

**Figure 5.**
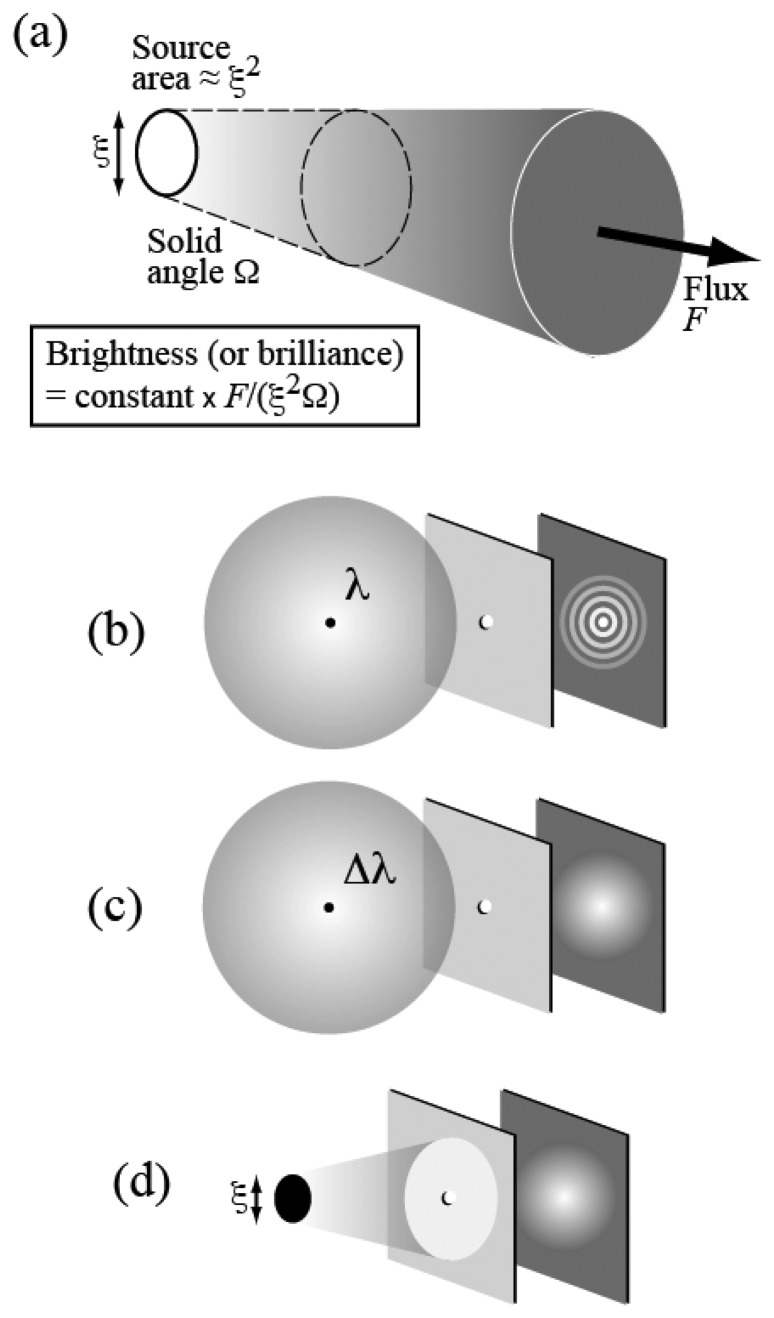
(a) Schematic definition of the essential source parameter “brightness” or “brilliance”. (b), (c) and (d) definition of time and spatial coherence by using diffraction by a pinhole.

In addition to being very intense, i.e. with a very large flux, a synchrotron source is also very bright. The high “brightness” or “brilliance” is the combined result of the large flux and of good geometric characteristics: a small emitting area and low angular divergence – see Figure 5a.

These parameters are not only entirely determined by the emission geometry of a single electron. In fact, the electrons in a storage ring circulate in bunches; each electron has a slightly different energy with respect to the others and travels along a slightly different trajectory. This contributes to the angular divergence of the emission. Furthermore, the differences between the electrons produce a finite transverse area of the bunch. This is the effective source area that must be used in the definition of the brightness or brilliance.

A bright source boosts the effectiveness and scope of many different x-rays techniques. Furthermore, high brightness is also associated to a high level of spatial coherence. The notion of coherence (Figure 5b) is quite fundamental in optics. The light, consisting of electromagnetic waves, can in principle produce wave-light phenomena such as interference or diffraction. In practice, however, these phenomena are rarely observed in everyday life. The reason is that to produce observable phenomena of this kind the light must possess a sufficient level of coherence.

Let us consider in fact the diffraction by a pinhole (Figure 5b). A point source emitting only one wavelength *λ* will always produce an observable diffraction pattern. However (Figure 5c), the emission of multiple wavelengths (or of a wavelength band, *Δλ*) will produce different patterns, one for each wavelength – and the superposition can wash out the diffraction effects. Likewise (Figure 5d), an extended source will act as a collection of point sources: the superposition of their patterns can wipe out the features of the individual patterns so that no diffraction is observable.

The notion of coherence thus includes two different aspects (1) “time” or “longitudinal” coherence: the emitted wavelength bandwidth *Δλ* must be sufficiently narrow; (2) “spatial” or “lateral” coherence: the source size *ξ* must be small enough -- and the angular divergence must be also limited. This second condition is also a condition for increasing brightness or brilliance as defined in Figure 5a. Synchrotron sources became brighter in the past 40 years, primarily because of the improvement of their geometric characteristics; as a byproduct, they also became more spatially coherent.

Special magnet devices called “undulators” and “wigglers” [7, 8] further enhance the brightness and coherence of modern synchrotron sources. Such devices use periodic arrays of magnets rather than a single bending magnet to produce the emission of synchrotron light. The resulting light possesses superior characteristics since the devices are optimized for the task of emitting it.

The historical improvement of brightness and coherence have made possible over four decades new experimental techniques or better versions of existing techniques. This applies specifically to the two analytical methods discussed here.

## 3. Photoemission Spectromicroscopy – Essential Background

Since the 1950s, experimental methods based on the photoelectric effect have been one of the major tools for chemical and physical analysis. Photoemission spectromicroscopy adds lateral resolution, so that the analysis can be performed on a scale now reaching the nanosize.

The conceptual background for photoemission-based methods [7, 8] is primarily provided by Einstein's hypothesis of the existence of photons. An electron in a solid or in a molecule becomes a photoelectron after absorbing a photon *h v* that increases its energy from the ground-state value *E_i_* to an excited value *E_i_* + *h v* above the barrier that keeps electrons from escaping into the vacuum. Thus, by capturing photoelectrons, measuring their energy and correcting it by *h v*, one can derive the ground state energy *E_i_*.

**Figure 6.**
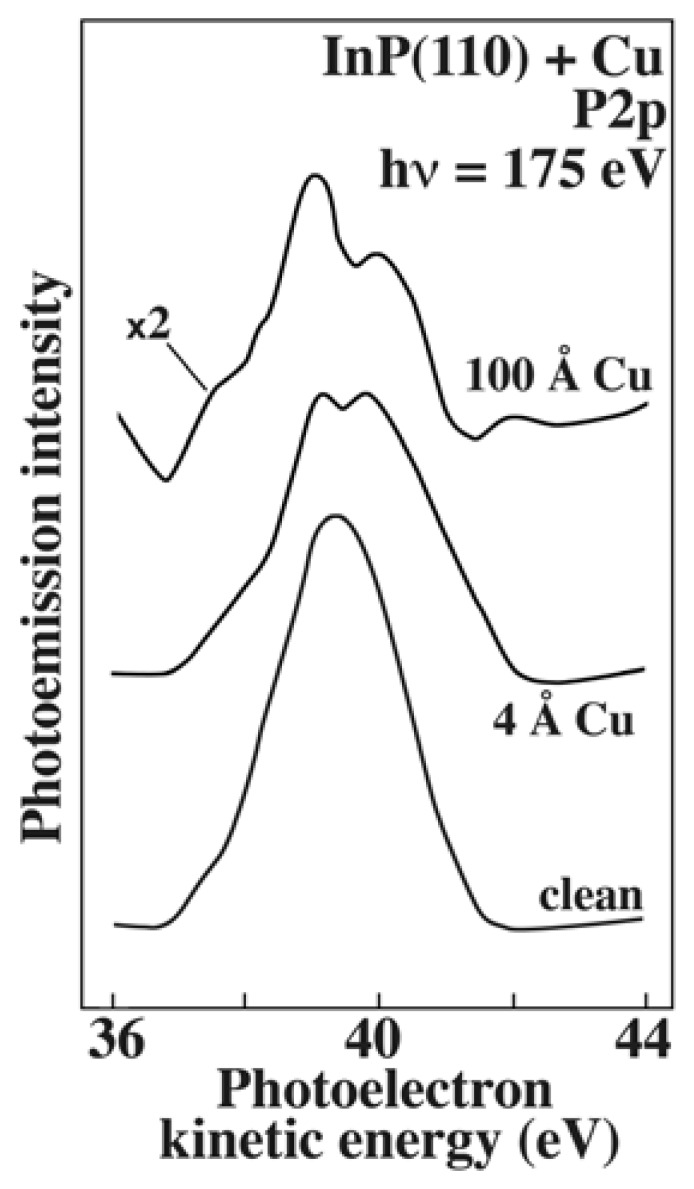
Photoemission spectra of a clean InP surface (bottom) and of the same surface covered by two different thicknesses of copper. The spectra show the P2p core level, revealing the presence of phosphorus and then the lineshape changes that reflect the modifications in the chemical bonding situation of the phosphorus atoms at the surface. Data from [13].

This makes it possible to analyze the electronic states of condensed-matter systems and therefore the properties of the corresponding chemical bonds. Figure 6 shows [13] one of the very many examples of this approach: the measured energy distribution of the electrons originating from the P1s core level. The simple detection of the core level peak reveals the presence in the system of the corresponding element – phosphorus in this case.

However, the information carried by spectra like those of Figure 6 goes well beyond the mere detection of an element. The study in [13] concerns the surface of an InP crystal: after being cleaned, it is progressively covered by copper. We see that after coverage the P2p peak becomes more complex and no longer corresponds to one single core-level energy of isolated atoms. This is due to the core-level energy perturbations caused by the charge distribution around the P atoms. Such perturbations are linked to the formation of new chemical bonds between P and Cu, different from the P-In bonds of the clean surface. Therefore, from the analysis of the core-level lineshape one can retrieve very valuable information on the chemical bond properties.

Historically [7, 8], the development of photoemission spectroscopy encountered several technical obstacles that retarded its real take-off from 1905 – the year of Einstein's theory later rewarded by the Nobel prize – to the 1950s. A major problem – as well as an excellent opportunity - was the surface sensitivity of the experiments. The escape depth of photoelectrons from solids is very short and the spectra are thus very sensitive to the surface conditions. Without being able to clean the sample surface and keep it uncontaminated under ultrahigh vacuum, photoemission spectroscopy is impossible: its real development started only after the advent of ultrahigh vacuum technology.

A second major problem was the signal level. To obtain a good signal, an intense source of high-energy photons is required: synchrotron light facilities thus played a fundamental role [7, 8]. Furthermore, the broad wavelength band of synchrotron light makes it possible to select the best photon energy and/or to perform photoemission experiments at different photon energies, enhancing both their flexibility and their reliability.

A third major problem in the development of photoemission techniques was the lack of lateral resolution. The chemical analysis could only look at the averaged properties of a surface area of the order of 1 cm^2^. It thus missed the wealthy of phenomena occurring on a smaller scale, including most biological mechanisms. This crucial limitation was removed by the advent in the late 1980s of photoemission spectromicroscopy [7, 8, 14, 15].

**Figure 7.**
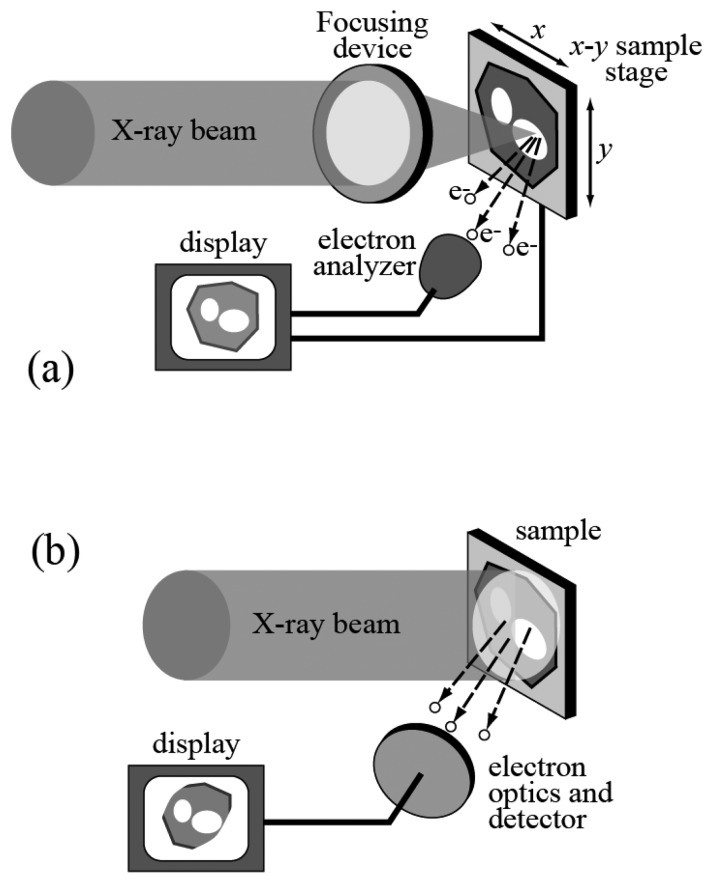
The two possible approaches to photoemission spectromicroscopy. In (a), lateral resolution is achieved by focusing the x-ray beam into a small spot and obtaining photoelectron spectra from the same sample area. An *x*-*y* stage can be used to scan the illuminated spot on the sample and obtain images. By selecting the photoelectron energy, such images can deliver specific microchemical information. In (b) the lateral resolution is achieved instead by using an electron optics system.

As the name suggests, this is the combination of photoemission spectroscopy and microscopy, i.e. high lateral resolution. Historically, high resolution was achieved [7, 8, 14, 15] with the two approaches outlined in Figure 7.

In the first case, the photon beam that stimulates the emission of photoelectrons is focused into a small spot. Thus, the photoemission spectra only carry information about that specific area. One can also scan the spot over a broader area, obtaining, for example, two-dimensional photoemission intensity “images” that carry fine chemical information.

**Figure 8.**
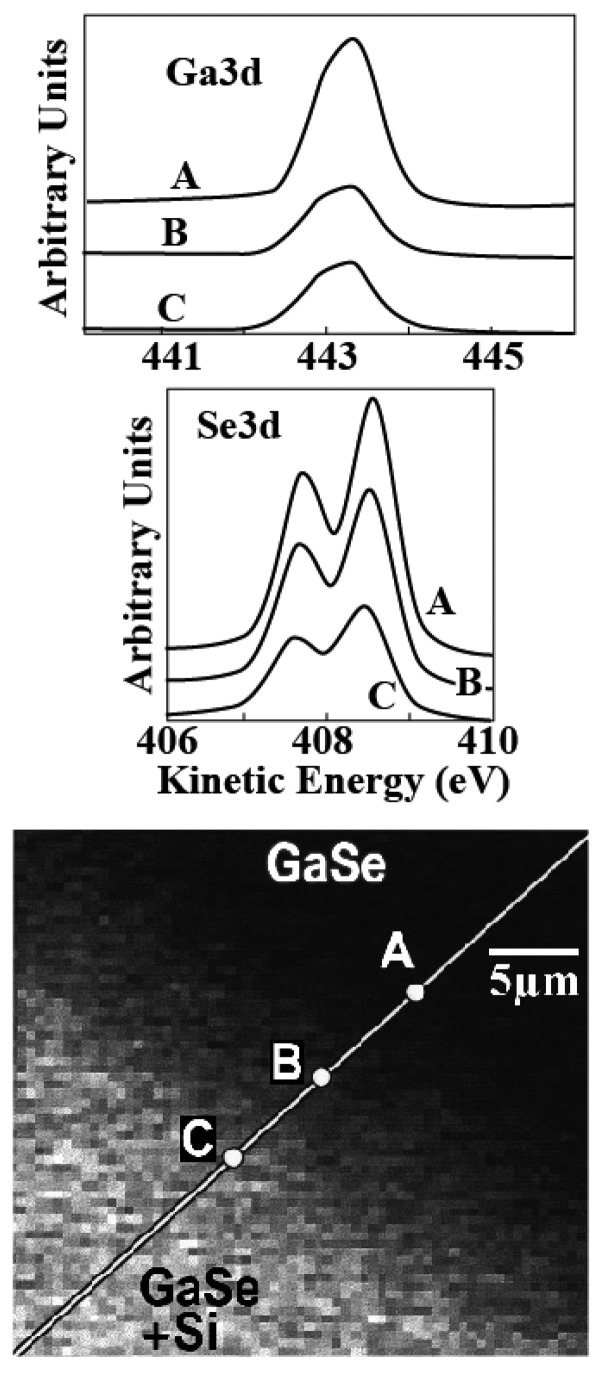
Top and middle: core-level photoemission spectra taken with the approach (a) of Figure 7; the peaks were obtained from a GaSe surface partially covered with Si. Bottom: image of the surface obtained by detecting the Si2p core-level photoemission peak intensity. Points A, B and C mark the positions where the Ga3d and Se3d spectra were taken. Note that the intensity of the Ga and Se peaks is lower in the Si-covered areas. Data from [16].

**Figure 9.**
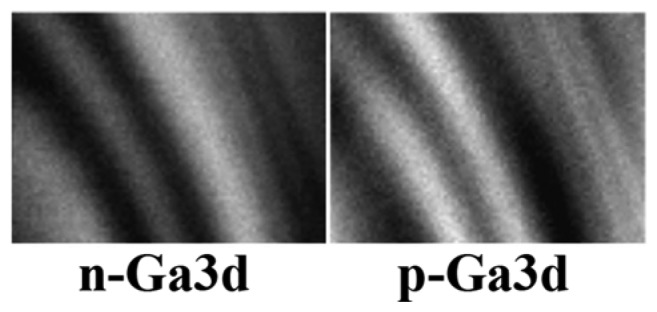
Photoemission intensity images obtained with approach (a) in Figure 7 by selecting photoelectron energies corresponding to the Ga3d core level in n-type and p-type GaAs. The complementary images show the transverse cross section of a complex GaAs structure with n-type and p-type layers. Data from [17].

Figures 8 and 9 shows two examples of this approach. In Figure 8, the information provided [16] is the presence and concentration of specific chemical element. The analyzed system is a cleaved substrate of GaSe partially covered with a Si overlayer. The Si-covered area is the brighter part of the bottom image. The top spectra show the Ga3d and Se3d core levels. It is clear that their intensity decreases on going from the Si-free area (point A) to the covered area (points B and C) – reflecting the coexistence of Ga and Se with the Si overlayer.

The bottom image of Figure 8 was obtained by measuring the Si2p core level intensity while scanning the illuminated area. Therefore, the image is a picture of the content of silicon. Indeed, it does correlates well with the weaker of the Ga and Se signals where Si is present.

Figure 9 is an example [17] of more detailed information than the mere presence of elements. In fact, both images refer to the same element, Ga, and to its 3d core level. The images were taken on the transverse cross section of a complex GaAs layered structure formed by both n-type and p-type layers. The local electrostatic potential changes between the n and p zones, and so does the Ga3d core-level energy and the corresponding photoemission peak position. Therefore, by selecting the spectral position one can obtain Ga3d photoemission intensity maps for n-type GaAs or for p-type GaAs. Since the n-type or p-type character is determined by the doping impurities, such maps reveal the impurity spatial distribution. This point is confirmed in Figure 9 by the fact that the n-type and p-type maps are complementary to each other.

The second spectromicroscopy approach of Figure 7 is not based on focusing the x-ray beam but on the use of an electron optics system to process the emitted photoelectrons. The experimental system thus becomes somewhat similar to an electron microscope -- except that the electrons are generated from the sample itself with the photoelectric effect. This makes it possible to obtain morphological information on the specimen and, by adequate energy filtering, microchemical information.

**Figure 10.**
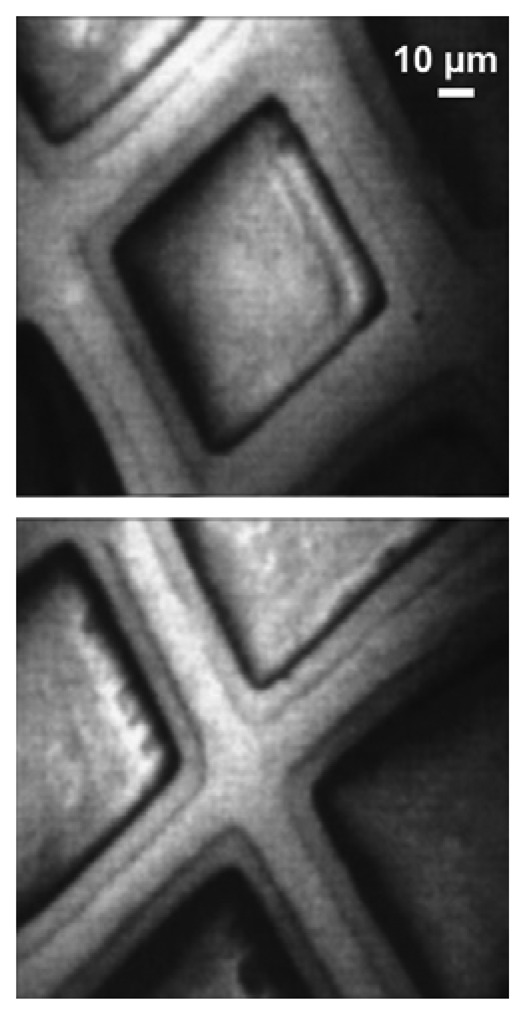
Images of a copper mesh obtained with approach (b) in Figure 7. Note the high lateral resolution. Data from [18].

**Figure 11.**
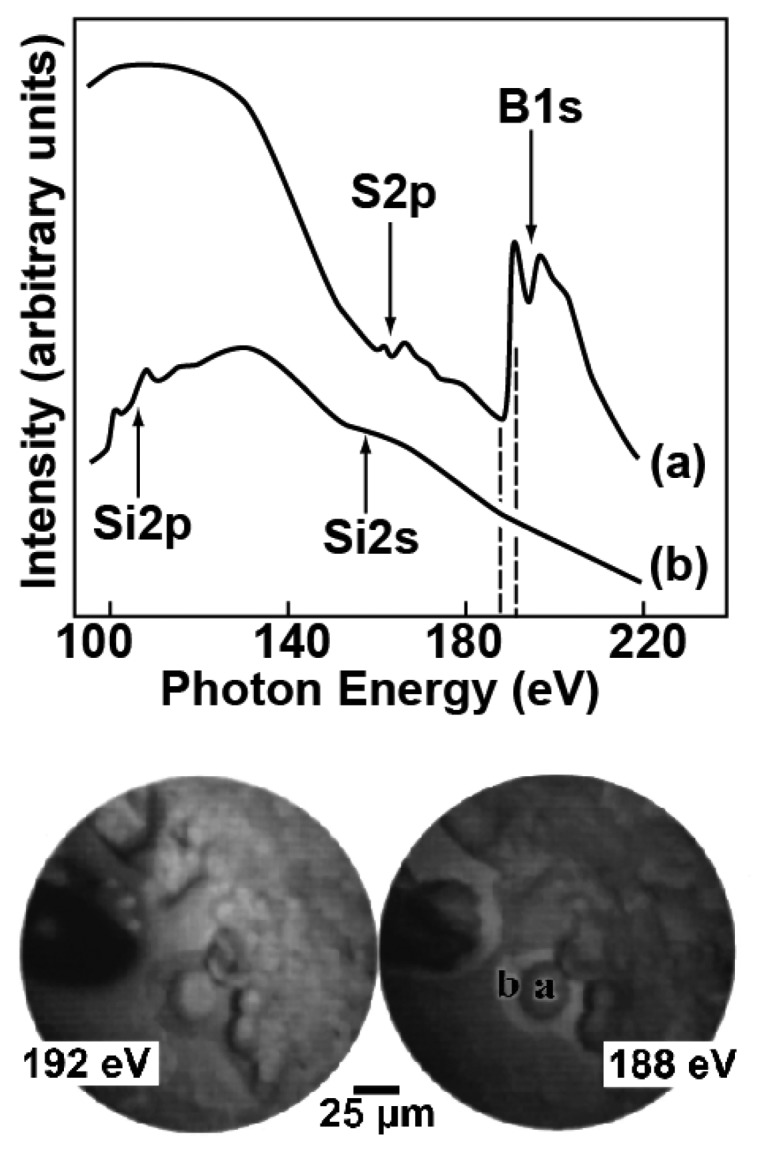
Top: surface optical absorption spectra obtained with the “partial yield” approach by Gudat and Kunz [19] and with high lateral resolution. The spectra were taken in the two areas “a” and “b” of the bottom images and correspond to a silicon substrate covered (“a”) or not (“b”) by BSH. The two images were obtained by exciting photoelectrons with two different photon energies right below and above the B1s threshold. Data from [18].

Figures 10 and 11 show two examples [18] of results obtained with this approach. In the case of Figure 10, images of a copper mesh were obtained by collecting photoelectrons of all energies after processing them with an electron-optics system. The images were obtained with the imaging spectromicroscope MEPHISTO [18] and the photoelectrons were produced by monochromatized photons from a Wisconsin Synchrotron Radiation Center (Aladdin) beamline.

Images like those of Figure 10 show the good lateral resolution of the MEPHISTO spectromicroscope – that reached in this case 50 nm but was subsequently surpassed by other instruments. However, such images do not immediately reveal the chemical analysis capabilities of the instrument. Such capabilities are illustrated by Figure 11: on the top, we see two photon absorption spectra obtained with an approach pioneered by Gudat and Kunz [19]: the photoemitted intensity due to photoelectron at all energies is scanned as a function of the photon energy. Without lateral resolution, this approach would measure the optical absorption coefficient of a wide area of the specimen surface. In Figure 11, the lateral resolution provided by the MEPHISTO electron optics was exploited to measure the surface absorption coefficient of small areas.

The two spectra of Figure 11 specifically concern a dried droplet of dodecahydro-dodecaborate (BSH) and were taken in two different specimen regions. We see large differences between the two curves, revealing in one case (“a”) a strong contribution from spectral features related to boron and sulphur absorption thresholds – whereas the spectrum “b” is dominated by the (substrate) silicon features. These findings correspond to the microchemical composition of the specimen, dominated by boron in areas where the dried BSH is present and by silicon elsewhere.

The two images at the bottom of Figure 11 further illustrate the microchemical capabilities of this approach. Each image was produced with photoelectron of all energies, but the exciting photon energies were different; specifically, the first photon energy, 188 eV, is right below the B1s absorption threshold in the “a” spectrum and the second photon energy, 192 eV, right above it. Therefore, the 192 eV image should emphasize, when compared to the 188 eV image, the boron contributions. We see indeed that the image intensity in areas “a” and “b” changes dramatically with the photon energy, with evidence of a strong boron presence in “a” and less boron in “b”. This complements and corroborates the results of the two spectra, taken in the same areas “a” and “b”.

Such results give a practical idea of the potential applications of photoelectron spectromicroscopy for nondestructive microchemical analysis -- in particular for art and/or historical specimens. Paradoxically, these opportunities were not so far widely exploited.

**Figure 12.**
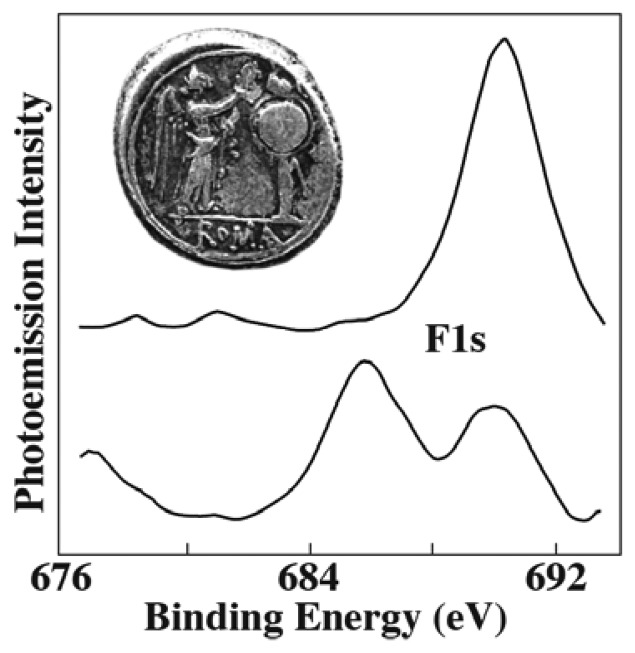
Use of photoelectron spectromicroscopy to study the microchemical composition of a Roman coin (the optical microscopy image). The local spectra taken after sputtering reveal the unexpected presence of fluorine below the surface. Data extracted from [20].

Figure 12 shows one of the few counterexamples [20]: photoemission spectra in the F1s spectral region were taken from small areas of a 211 B.C. Roman silver coin. Sputtering made it possible to probe regions below the initial surface. The two spectra in Figure 12 were taken, with moderate lateral resolution, in the coin areas corresponding to the arm and to the shield.

Whereas fluorine was not detected before sputtering, its presence below the surface is evident from the spectral results of Figure 12. This was an unexpected discovery since fluorine had never been found in silver coins. The result triggered a number of intriguing hypothesis about the possibility of empirical addition of fluorite by the Romans, using a metallurgical practice subsequently lost through history.

## 4. Elements of Coherent-X-Ray Microradiology

Radiology is the oldest, and by far the most widespread application of x-rays. The bulk of its use is of course in medical diagnostics – but other applications are also quite important. Both medical and non-medical applications are primarily based on absorption contrast: the morphology of the object is revealed by the differences in the x-ray absorption coefficient of its components.

Absorption is typically very weak for x-rays and this is both the key to the success of radiology and its main technical limitation. Weak absorption enables x-rays to penetrate into the objects and to analyze their otherwise invisible inner parts. However, weak absorption also means weak contrast between different parts. This limitation is quite evident in the case of soft tissues whose chemical composition is similar. The consequences are very negative: for example, coronary angiography requires the invasive injection of contrast agents and cannot be used as a screening technique for heart diseases. Likewise, screening of breast cancer by mammography remains controversial because of the large x-ray dose and in spite of the clear advantages of early detection. Absorption, however, is only one aspect of the interaction of x-rays with matter. Could other interaction mechanisms [7, 8, 21] enhance the contrast in radiology? Let us look at the broader picture by considering for simplicity a plane, linearly polarized (along the *y*-axis) monochromatic x-ray wave propagating along the *x*-axis – whose electric field in vacuum is *E_y_*(*x, t*) = *E_o_*exp[i(*kx* – *ωt*)] (*k* = 2π/*λ* is the wavenumber, *λ* the wavelength and *ω* the angular frequency).

In a material, this wave function becomes *E_y_*(*x, t*) = *E_o_*exp[i(*nkx* − *ωt*)], where *n* is the (frequency-dependent) complex refractive index, *n*(*ω*) = 1 − *δ*(*ω*) + i*β*(*ω*). The imaginary part i*β*(*ω*) gives an exponential factor with a real negative exponent proportional to *x*. This is of course the attenuation factor that describes absorption.

The real part 1 − *δ*(*ω*) equals the conventional refractive index and gives instead an imaginary-exponent exponential factor. The exponent is the phase of the wave and accounts for phenomena like refraction, interference and diffraction. For the x-rays used in radiology, both absorption and refraction are quite limited. Therefore, the absorption coefficient is very small and the conventional refractive index very close to unity. In other words, both *β*(*ω*) and *δ*(*ω*) are small.

The absorption factor *β*(*ω*) is the foundation of conventional radiology. Could, however, the factor *δ*(*ω*) and the corresponding mechanisms also contribute to the image contrast? In principle yes, but under certain conditions.

Refraction, diffraction and interference phenomena can be observed – and contribute to the image contrast - if the x-ray beam is sufficiently directional and monochromatic, i.e. with adequate levels of lateral and longitudinal coherence. Standard radiology is performed with broadband, large-size anode sources emitting over a large angular range: such sources are not coherent. Hence, *δ*(*ω*) plays in practice no role in conventional radiology whose contrast is fully due to absorption.

Synchrotron sources change the picture by providing a high level of coherence. The corresponding contrast enhancement radically changes the scope and performances of radiology. In particular, coherent-x-ray radiology can now work on a microscopic scale and in real time -- the present resolution levels being <1 micron for space and <1 millisecond for time.

**Figure 13.**
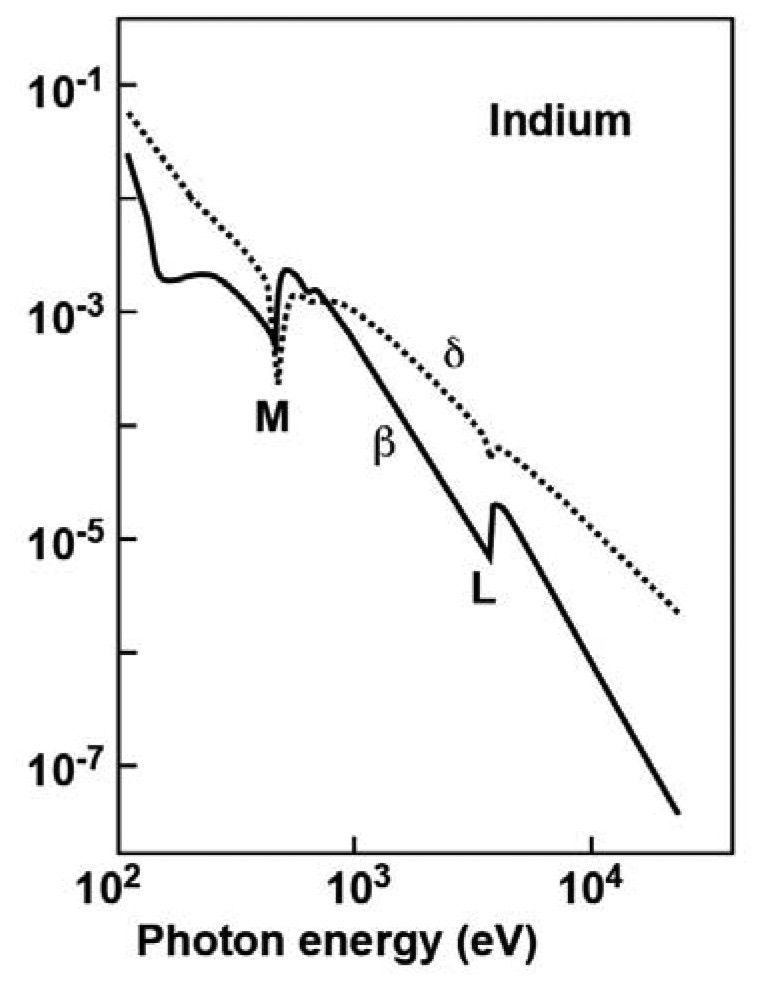
Plots of the *β* and *δ* factors as a function of the photon energy for indium. The features corresponding to the L and M thresholds are marked.

Figure 13 shows typical plots (in the case of indium) of the *β* and *δ* factors as a function of the photon energy in the x-ray spectral range, exhibiting several features of interest to our discussion. We see that both *β* and *δ* are indeed very small. Note, however, two additional important facts. First, in most of the spectral range *δ* is not as small as *β*, suggesting that the corresponding contrast mechanisms can indeed play a prominent role.

Second, at each core level threshold (the L and M shell thresholds in Figure 13) both *β* and *δ* change rapidly; the change of *β* is explained by the activation of optical excitation processes from the core level. The changes in *δ* are a consequence of the links between different optical phenomena (absorption, refraction, diffraction, interference) due to fundamental physical properties and expressed by the so-called “Kramers-Krönig relations” [8].

The changes in *β* near the absorption threshold of a given element can be used to identify element-specific features in absorption-contrast images. This is implemented by comparing images taken at photon energies right above and right below the threshold. The corresponding changes in *δ* indicate that a similar possibility exists for coherence-based radiology: the first tests of this approach [22] were quite successful.

We can now discuss the basic issue: how can the real part of the complex refractive index contribute to image contrast? In general terms, it determines the phase of the x-ray wave whereas the imaginary part determines its intensity. The combination of the two effects produces the equivalent of on-line Gabor holograms [8]. One could thus imagine how to reconstruct such holograms and obtain three-dimensional images of the object.

**Figure 14.**
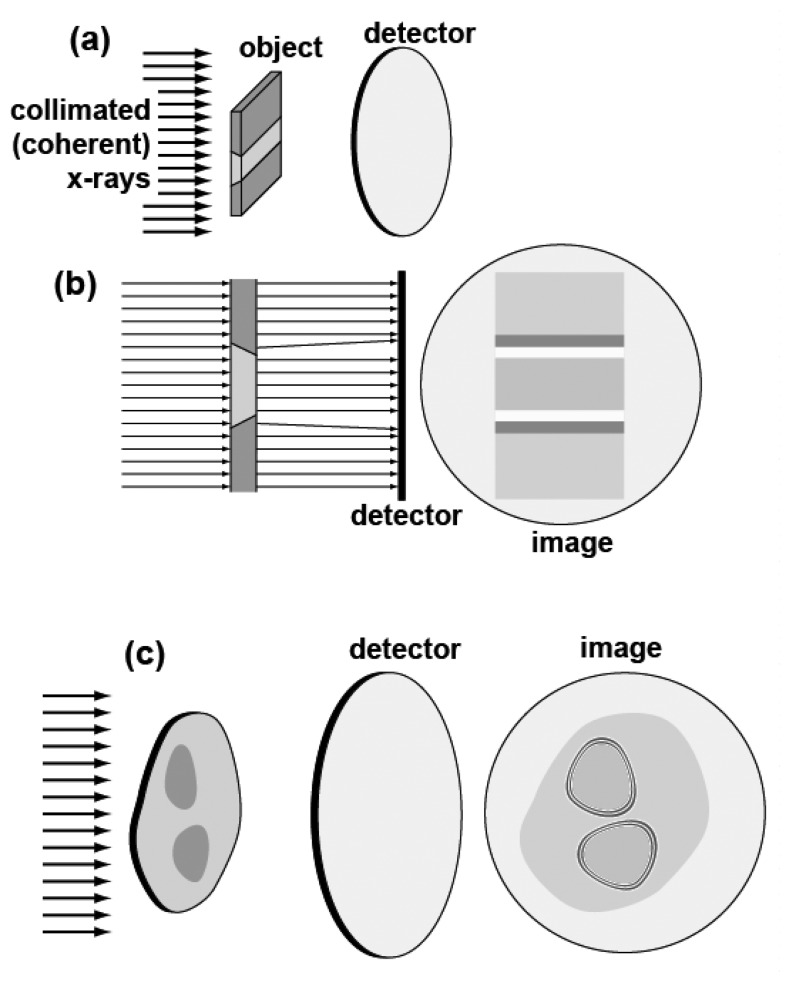
Schematic explanation of the edge enhancement mechanisms in radiological images that are produced by coherent x-rays and are due to the real part of the complex refractive index. (a) and (b) show edge enhancement due to refraction and (c) a Fresnel-like diffraction mechanism [23, 24].

This approach, however, is too complicated for most of the problems treated with coherent-x-ray radiology. The best strategy is to limit the retrieved information to what is really relevant to the problem under investigation – thus simplifying the data interpretation. In order to understand the simple conceptual background, let us consider [8, 23, 24]Figure 14.

The top parts, Figures 14a (perspective view) and 14b (side view), illustrate the contribution to the contrast related to the real part of the refractive index that are due to simple refraction. The object consists of different regions with two different values of the (real part of the) refractive index. Such regions are separated by slanted edges. The collimated x-ray beam is not deviated by refraction except by the slanted edges.

Refraction deviates the x-rays in the region of the edges, producing for each edge a characteristic white-dark double fringe in the image. These features strongly enhance the visibility of the edges whereas mere absorption would make the different regions almost indistinguishable.

The bottom part shows that even in the absence of slanted edges between regions with different values of the (real part of the) refractive index there is edge enhancement. The mechanism in this case is equivalent to the classical phenomenon of Fresnel diffraction by an opaque object illuminated by visible light – that produces a whole series of fringes at each edge [23]. In the case of x-rays, the objects are not opaque but have different refractive index (real and imaginary parts). But the effect is still the creation of a series of fringes that enhance the edge visibility.

**Figure 15.**
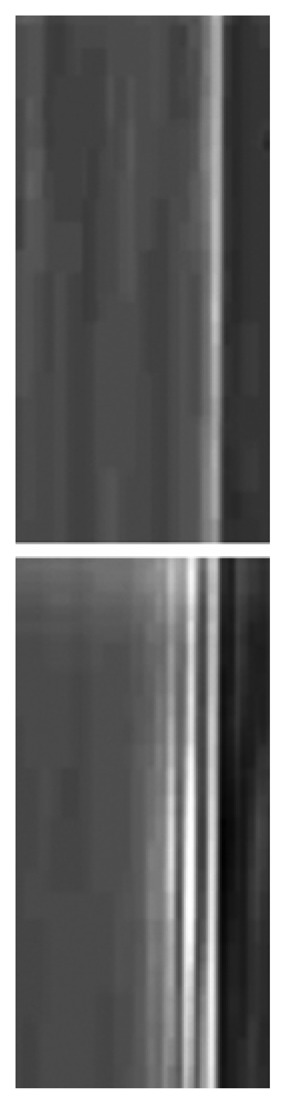
Images of the same edge of an optics fiber taken with different experimental geometries [23, 24]. In the top image, the edge enhancement is due to refraction effects whereas in the bottom case the mechanism is diffraction.

In practice, both of the above edge-enhancing phenomena – refraction and diffraction - coexist in an image. However, it was demonstrated [23, 24] that by changing the experimental geometry either one or the other can be enhanced – see Figure 15. As we already mentioned, in most cases it is preferable to enhance the refraction-based edge enhancement that is simpler to interpret – and de-emphasize the diffraction fringes that correspond to a hologram but are more complicated for a simple inspection of the image.

What are the conditions [23, 24] for observing the edge enhancement? Obviously, the x-ray beam must be collimated, i.e. spatially coherent – since otherwise penumbra effects wash out the edge enhancement. Furthermore, the x-ray beam must be reasonably monochromatic – i.e. longitudinally coherent - to avoid excessive fringe broadening that can also wash out the edge-enhancing fringes. Therefore, x-rays with a sufficient level of both spatial and longitudinal coherence are needed.

The question, however, is: how much coherence is necessary? As a matter of fact, not very much. Let us start with the edge enhancement by a series of diffraction fringes. Concerning longitudinal coherence, a relative wavelength bandwidth Δλ/λ ≈ 1 is already sufficiently small to see the fringes [8, 23]. This is not a stringent requirement; in fact, this level of monochromaticity is automatically provided by different kinds of synchrotron sources without any additional spectral filtering. As to lateral coherence, a source size of the order of 100-200 μm is sufficient [23]. This again is not a stringent requirement, and it is automatically met by most present synchrotron sources (but least only in the vertical direction in the case of bending magnets).

We shall not turn our attention to edge enhancement by refraction. There is no condition for longitudinal coherence and a simple analysis [24] shows that the requirements for lateral coherence are once again rather mild.

In essence – and contrary to a common belief – refractive-index radiology does not require a very advanced synchrotron source nor a very sophisticated beamline. This realization is important since it implies that its practical implementation is not extremely difficult. Furthermore, the technique does not require the object to be kept in vacuum: this is of course a big advantage in the study of artistic and/or historical specimens. The limited requirements for longitudinal coherence make it possible, in particular, to avoid the use of monochromators and the related loss of x-ray intensity [23, 24]. This in turn can be exploited to enhance the spatial and time resolution -- and perform real-time analysis on a microscopic scale.

## 5. Some Practical Examples of Coherent-X-Ray Radiology

**Figure 16.**
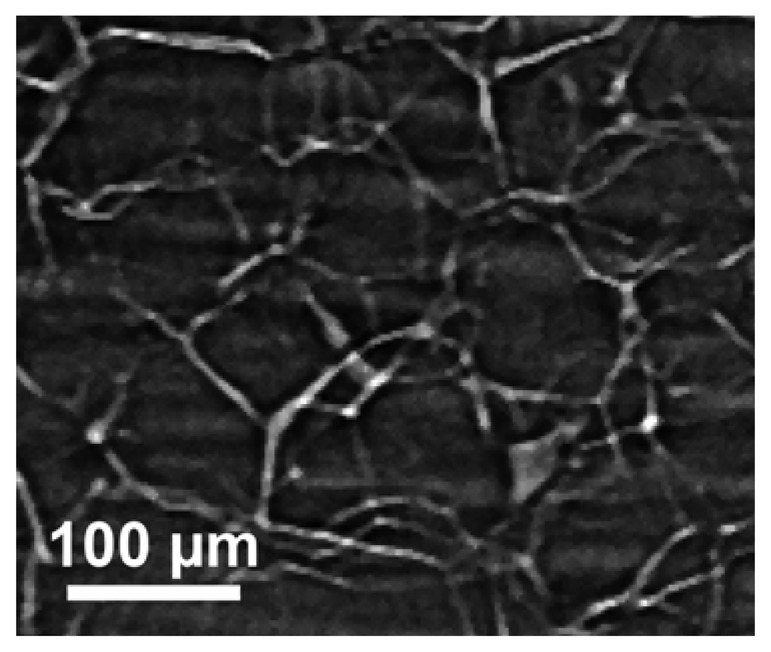
Refractive index radiology reveals in detail the edges of grain boundaries in a polycrystalline polypropylene [data from Ref. 25]. The grain boundaries are visible as white areas without using any decoration.

The purpose here is to stimulate the attention of researchers on the opportunities offered by radiology with coherent x-rays for nondestructive analysis. We do not intend, on the other hand, to present an extensive review of recent results. Figure 16 illustrates the capabilities of the simplest approach to coherent-x-ray radiology. The edge enhancement reveals in this case [25] minute details of a materials science specimen – and in particular, the location of grain boundaries without any decoration procedure that could modify the specimen properties. The visibility of small details is quite evident and even more remarkable if one considers the absence of any artificially added contrast agent.

**Figure 17.**
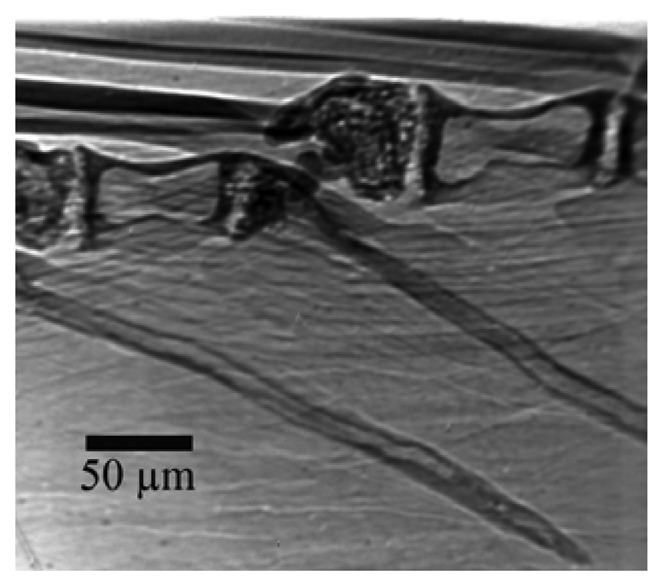
Refractive index microradiograph of a detail of a live 5-mm aquarium fish (data from Ref. 25).

Figure 17 shows an example of the applications to live biological specimens. We see here [25] microscopic details of a very small fish in water. The enhanced contrast makes it possible to reduce the x-ray dose and the subsequent possible damage to live specimens. The same point applies to delicate art and historical specimens whose damage, even limited, must be absolutely avoided.

The above results were both obtained in the “projection” mode of radiology, i.e. by taking only one image and not using any image reconstruction software. The rapid image taking, however, makes it possible to obtain thousands of images in a few seconds. This can be exploited to implement coherent-x-ray radiology in the tomographic mode [26]. After taking images of the object from many different directions, suitable algorithms are applied in this case for image reconstruction. The results are diversified and can be quite sophisticated.

**Figure 18.**
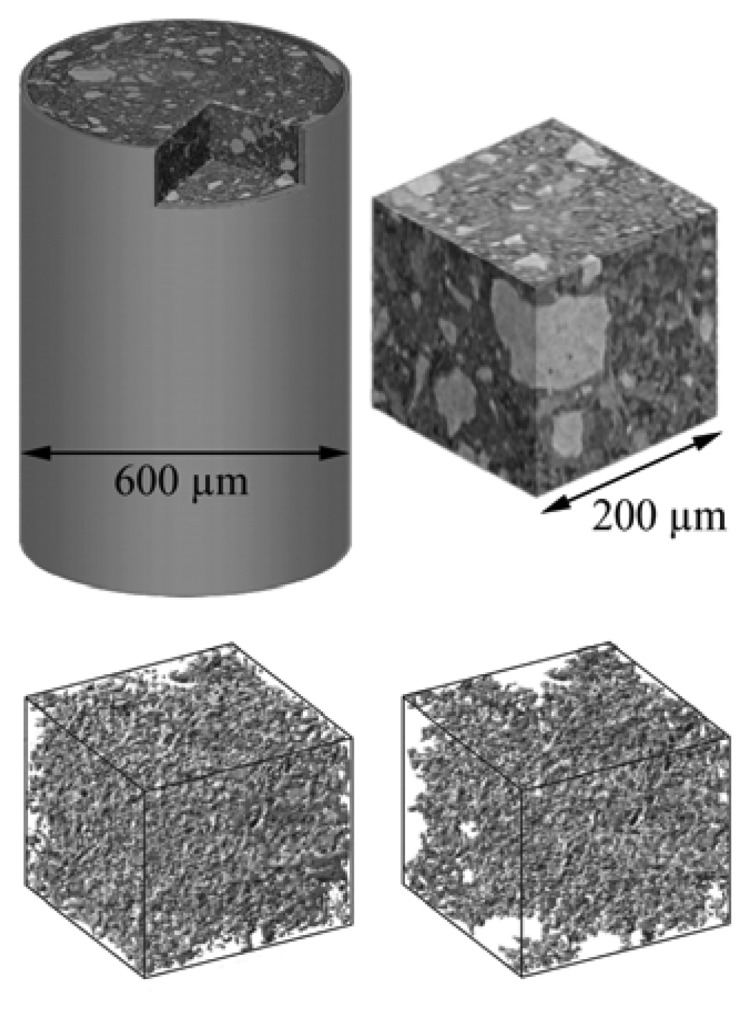
Refractive index microradiology implemented in the tomographic mode: computer-reconstructed images derived from a large number of projection radiographs. The top images (data from Ref. 27) were obtained for a 1 day old cement sample and show a reconstructed rendering of the entire sample as well as that of a specific volume. The bottom images – referring to a 3 day old sample - show how computer processing can yield reconstructed images of the pores (bottom left) and of the fraction of the pores that are interconnected or “percolating”.

Figure 18 shows an example in materials science that could be also interesting for archaeology. The chemical evolution of concrete specimens was analyzed [27] with the specific objective to study the network of pores and their connectivity – that could influence the continuing exposure to the atmosphere and the consequent chemical reactions. The figure shows how sophisticated this approach can become in delivering information about the microscopic inner structure of objects without damaging them.

**Figure 19.**
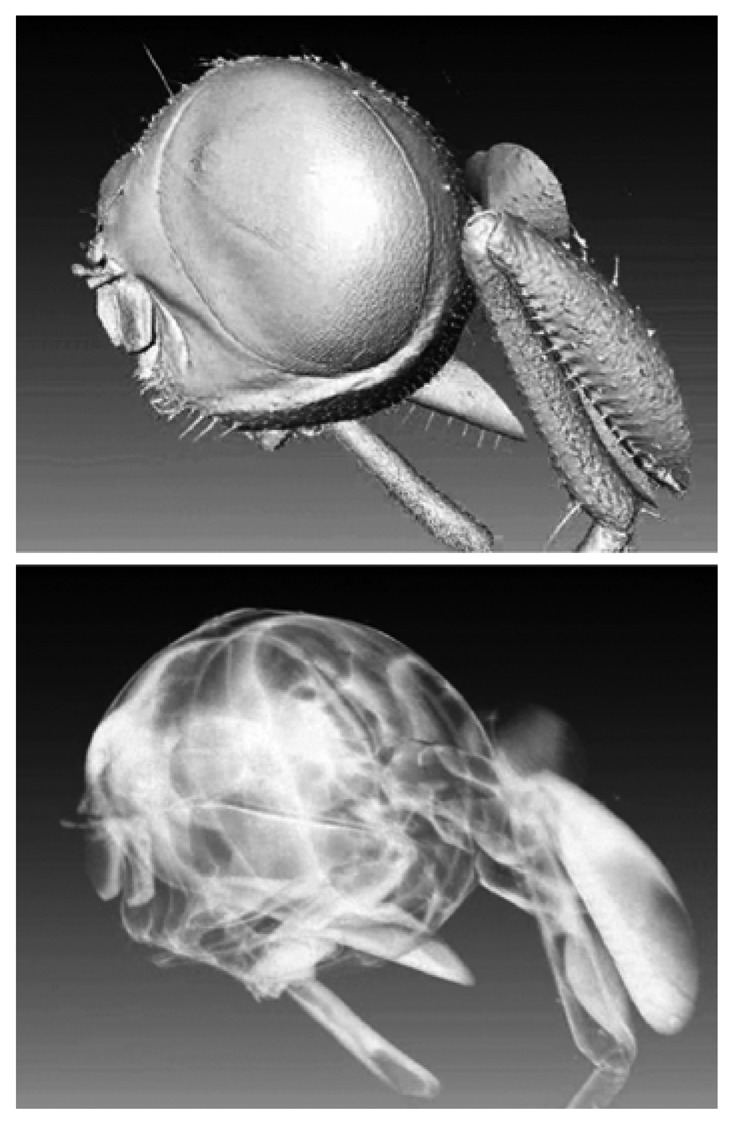
Refractive index microtomography applied to a biological specimen: reconstructed images of the head of a housefly. At the top, a volume image; at the bottom, a “peeled off” rendition of the same specimen.

Tomography can be also applied to organic and animal specimens. Figure 19 shows a rather stunning example in which the object could be “peeled off by the computer processing to reveal inner details on a submicron scale.

**Figure 20.**
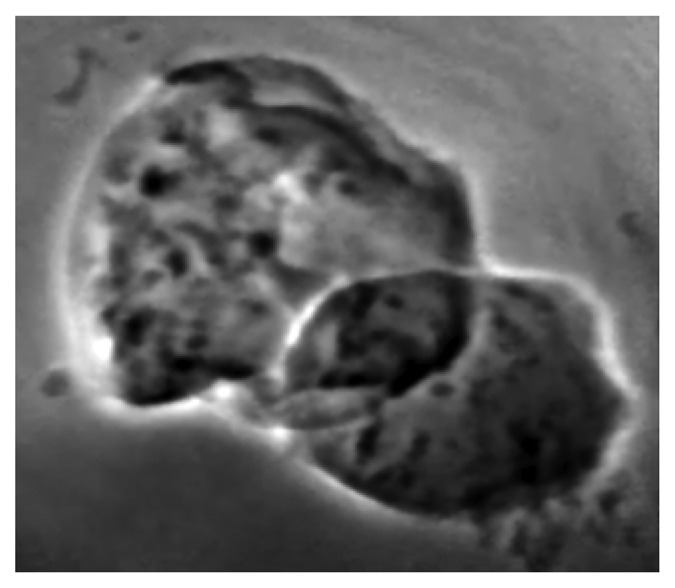
Refractive index micrograph of EMT (Epithelial-Mesenchymal Transition cells with gold nanoparticles.

Finally, Figure 20 illustrates the use of high spatial resolution to investigate biological specimens down to the cellular level [28]. Quite recently, even this benchmark level of resolution was passed and now the studies can investigate subcellular details in animal tissues.

## 6. Concluding Remarks

Synchrotron-based techniques are increasingly present in the nondestructive testing of art and historical objects and in general in the effort to preserve cultural heritage. However, such efforts still use only a subset of the wide variety of techniques relying on synchrotron sources. Other techniques in this arsenal could be quite useful in this context.

We specifically described photoelectron spectromicroscopy and coherent-x-ray radiology illustrating their remarkable capabilities for structural and chemical analysis on a microscopic scale and in real time. These are no longer techniques under development but mature instruments that find widespread use in many areas. Their extension to the preservation of cultural and historical heritage would be quite desirable.

It should be noted that the techniques are not yet reaching their basic performance limitations. Further improvements are possible as far as spatial and time resolution are concerned as well as for the finesse of the chemical analysis. The forthcoming implementation of accelerator-based x-ray lasers [8] is likely to contribute to this evolution and should thus be carefully followed by the potentially interested scientists.
